# Chemopreventive Effects of *Citrus depressa* Leaf Extract Through Nrf2 Pathway Activation and Epigenetic Modulation

**DOI:** 10.3390/biomedicines14040813

**Published:** 2026-04-02

**Authors:** Hsin-Yu Chiang, Ssu-Han Huang, Tien-Yuan Wu, Yen-Chen Tung, Yung-Lin Chu, Hsiao-Chi Wang, Guor-Jien Wei, Zheng-Yuan Su

**Affiliations:** 1 Department of Bioscience Technology, Chung Yuan Christian University, Taoyuan City 320314, Taiwan; 2 School of Pharmacy, Taipei Medical University, Taipei City 110301, Taiwan; 3 Department of Food Science, National Ilan University, Yilan County 260007, Taiwan; 4 Department of Food Science, National Pingtung University of Science and Technology, Pingtung County 912301, Taiwan; 5 Department of Beauty Science, National Taichung University of Science and Technology, Taichung City 404336, Taiwan; 6 Institute of Food Safety and Health Risk Assessment, National Yang Ming Chiao Tung University, Taipei City 300093, Taiwan

**Keywords:** carcinogenesis, citrus, epigenetics, Nrf2, leaf

## Abstract

**Background/Objectives**: Many chronic diseases, including cancer, can be developed in conjunction with excessive intracellular oxidative stress and persistent inflammation. The importance of preventive strategies is highlighted by the potential of phytochemical interventions to mitigate these diseases. The purpose of this study was to investigate how *Citrus depressa* leaf (CDL) extracts can prevent 12-*O*-tetradecanoylphorbol-13-acetate (TPA)-induced carcinogenesis in JB6 P+ mouse skin epidermal cells. **Methods**: CDL extracts were prepared and characterized for their phenolic and flavonoid contents. Effects of the potent extract on cell viability, TPA-induced colony formation, intracellular reactive oxygen species (ROS) levels, and nuclear factor erythroid 2–related factor 2 (Nrf2)-related protein and mRNA expression, mediated by epigenetic modifications, were evaluated in JB6 P+ cells. **Results**: Both the water extract (CDL-WE) and the 95% ethanol extract (CDL-95EE) contain abundant flavonoids that inhibit TPA-induced cell transformation and colony formation without minimal cytotoxicity. Mechanistic studies indicated that CDL-95EE increased the gene expression of Nrf2-related detoxification and antioxidant enzymes, such as UDP-glucuronosyltransferase 1A (UGT1A) and heme oxygenase-1 (HO-1), and decreased intracellular ROS accumulation. Furthermore, CDL-95EE reduced the expression of epigenetic modifiers, including DNA methyltransferases (DNMTs) and histone deacetylases (HDACs), suggesting involvement in epigenetic regulation. **Conclusions**: These findings indicate that CDL, an agricultural by-product, may be useful in cancer prevention through antioxidant and epigenetic mechanisms.

## 1. Introduction

Cellular oxidative stress results from decreased antioxidant levels and increased free radical production. The human body generates ROS and free radicals due to endogenous and external factors, but, fortunately, the antioxidant defense system prevents oxidative damage [1]. Endogenous antioxidant enzymes such as glutathione peroxidase (GPx) and superoxide dismutase (SOD), as well as nonenzymatic antioxidants, including glutathione (GSH), vitamin C, and vitamin E, are used under normal conditions [2]. The accumulation of excessive free radicals can lead to oxidative stress when antioxidant defense mechanisms are exhausted, resulting in oxidative damage to DNA, proteins, and other cellular components [1]. The accumulation of oxidative damage can result in acute or chronic cell injury or persistent inflammatory responses, ultimately leading to the development of diseases such as cancer [3]. The activity of nuclear factor erythroid 2–related factor 2 (Nrf2) is regulated by oxidative stress, as it is a transcription factor that is highly sensitive to redox changes [4]. Nrf2 is sequestered in the cytoplasm by binding to Keap1 under normal conditions, but when cells are exposed to oxidative stress, it dissociates from Keap1 and moves to the nucleus [4]. It binds to antioxidant response elements (AREs) and induces the expression of downstream genes involved in antioxidant and detoxification processes [5]. These genes encode superoxide dismutase (SOD), glutathione peroxidases (GPx), peroxiredoxins, heme oxygenase-1 (HO-1), UDP-glucuronosyltransferase (UGT), glutathione *S*-transferase (GST), and NAD(P)H:quinone oxidoreductase-1 (NQO1) and other enzymes [5]. The increased antioxidant capacity of cells decreases oxidative stress and aids in cancer prevention [6].

Epigenetics is defined as the control of gene expression through mechanisms that do not change the DNA sequence. These mechanisms, including DNA methylation, histone modification, and microRNA actions, can lead to differential gene expression [7]. DNMTs are typically responsible for DNA methylation and occur primarily at CpG dinucleotides [8]. Methylation of a DNA region decreases its transcriptional efficiency, leading to reduced protein production and gene suppression [8]. The enzymes responsible for histone modifications include histone acetyltransferases (HATs), histone deacetylases (HDACs), and histone methyltransferases (HMTs) [9]. HATs loosen the structure of the chromatin to promote transcription, while HDACs compact the chromatin to suppress transcription [9]. The FDA has approved several drugs that inhibit HDACs and DNMTs for use as chemotherapeutic agents at present [10]. Furthermore, research has shown that epigenetic mechanisms mediated by phytochemicals can be used to prevent cancer. Sulforaphane and isothiocyanate are effective inhibitors of HDACs and DNMTs, thereby reducing methylation of the Nrf2 promoter and increasing Nrf2 mRNA expression [11]. Other natural cancer-preventive compounds, such as urosolic acid, curcumin, and tocopherols, have been found to regulate Nrf2 gene expression through epigenetic mechanisms [12].

Lime leaf extracts have been proven to be promising for use in health supplements, and kaffir lime leaves are recognized for their high total phenol and flavonoid content [13,14]. Essential oils extracted from lemon, kaffir lime, and lime leaves have shown potential antioxidant and antidiabetic effects [15]. Bitter orange, bergamot, and kaffir lime leaf extracts also exhibit antioxidant and anticancer effects [16,17,18,19]. Furthermore, cosmetic products already incorporate derivatives of sweet orange and bergamot leaves [20]. These findings, as well as recent reviews on other Rutaceae herbs, such as *Ruta graveolens*, indicate that Rutaceae plants are essential sources of bioactive phytochemicals with beneficial and potentially adverse effects, which need to be carefully characterized [21]. *Citrus depressa* Hayata, a fruit of the family Rutaceae, is also known as Shiikuwasha in both Japan and Taiwan for its strong, sour aroma [22]. *C. depressa* juice and peel extracts, which are known for their biological properties, and polymethoxyflavones (PMFs), such as nobiletin and tangeretin, possess anti-inflammatory properties and can prevent cancer [23,24,25].

Compared with other citrus peels and leaves that have been more extensively investigated, *C*. *depressa* leaves (CDL) also exhibit a distinct phytochemical profile. However, the chemopreventive potential of CDL, particularly through epigenetic modulation of Nrf2 signaling, remains largely unexplored. Investigating the cancer chemopreventive capacity of CDL could therefore not only add value to this underutilized agricultural by-product but also identify a unique source of Nrf2-activating and epigenetically active phytochemicals. In this study, we first used extraction solvents with different polarities (water, 95% and 70% ethanol, methanol, and ethyl acetate) to comprehensively characterize the flavonoid and phenolic composition of CDL and to relate solvent-dependent composition to extraction efficiency. We then selected CDL water extract (CDL-WE) and 95% ethanol extract (CDL-95EE) for further in vitro evaluation in mouse JB6 P+ skin epidermal cells to determine whether these extracts enhance cellular antioxidant capacity and inhibit TPA-induced cell transformation. In addition, we examined the relationship between CDL-95EE and Nrf2, and their downstream detoxifying and antioxidant enzymes, through transcriptional activation and epigenetic regulation, focusing on changes in DNA methylation and histone deacetylase expression.

## 2. Materials and Methods

### 2.1. Reagents and Materials

Dr. Guor-Jien Wei, from the Institute of Food Safety and Health Risk Assessment at National Yang Ming Chiao Tung University (Taipei, Taiwan), kindly provided compounds as follows: 7-hydroxy-3′,4′,5,6,8-pentamethoxyflavone, 4′-hydroxy-5,6,7,8-tetramethoxyflavone, 3′,4′,5,7-tetramethoxyflavone, sinensetin, 3′,4′,5′,5,6,7-hexamethoxyflavone, 4′,5-dihydroxy-6,7,8-trimethoxyflavone, nobiletin, tangeretin, 5-hydroxy-3′,4′,6,7,8-pentamethoxyflavone, and 5-hydroxy-4′,6,7,8-tetramethoxyflavone. Additional reagents and chemicals were obtained as follows: dimethylsulfoxide (DMSO), sodium bicarbonate (NaHCO_3_), 12-*O*-tetradecanoylphorbol-13-acetate (TPA), Basal Medium Eagle (BME), bacteriological agar, 99.8% ethanol, quercetin, caffeic acid, catechin and Folin–Ciocalteu phenol reagent from Sigma-Aldrich (St. Louis, MO, USA); acetonitrile from Aencore (Box Hill, VIC, Australia); Fetal bovine serum (FBS), penicillin-streptomycin, trypsin, gentamycin, glutamine, Minimum Essential Medium (MEM) and Dulbecco’s phosphate-buffered saline (DPBS) from Thermo Fisher Scientific (Waltham, MA, USA); the Cell Titer 96^®^ Aqueous One Solution Cell Proliferation (MTS) assay kit from Promega (Madison, MI, USA); methanol, 95% ethanol, and rutin from Echo Chemical (Miaoli, Taiwan); apigenin, luteolin, and myricitrin from ChemFaces (Wuhan, China); gallic acid, myricetin, synephrine, naringenin, and hesperidin from TCI (Tokyo, Japan); neohesperidin from Tauto Biotech (Shanghai, China); and 3′,4′-dimethoxyflavone from ALFA Chemistry (Ronkonkoma, NY, USA).

### 2.2. Preparation of CDL Extracts

Fresh *C. depressa* leaves (CDL) were collected from Pingtung, Taiwan, and only healthy, mature leaves without visible damage were selected. A voucher specimen has been deposited in the laboratory herbarium of Chung Yuan Christian University under the code CYCU-CD-001. As previously mentioned, the extraction methods were used to obtain bioactive compounds [26]. The CDL was washed and dried at 50 °C until it reached a constant weight, then ground into powder. To achieve a homogeneous fine powder, the powdered leaves were sieved through a 40-mesh sieve through a solid-based sieving process. For extraction, 1 g of leaf powder was mixed with 30 mL of each solvent, and the extraction with organic solvents (95% ethanol, 70% ethanol, methanol, and ethyl acetate) was performed at a controlled room temperature of approximately 25 °C for 24 h under gentle agitation, whereas the water extract was prepared by mixing with boiling water for 5 min. After vacuum filtration, the organic solvent and water filtrates were freeze-dried to obtain solvent-free extracts, respectively.

### 2.3. Determination of Total Phenolic Content (TPC)

The Folin–Ciocalteu method was used to determine TPC, with gallic acid as the standard [27]. The extracts were dissolved in 95% methanol at a concentration of 5 mg/mL, and standard solutions were prepared. Fifty μL of each extract or standard solution was added to 100 μL of the Folin–Ciocalteu reagent, followed by 400 μL of 700 nM Na_2_CO_3_. After incubation for 60 min, 200 μL of the mixture was transferred to a 96-well microplate and the absorbance was measured at 765 nm using a microplate reader (Synergy HT, BioTek, Winooski, VT, USA). The standard curve equation for gallic acid was used to determine the TPC of the extracts, expressed as gallic acid equivalents (GAE) per gram of dried extract.

### 2.4. Determination of Total Flavonoid Content (TFC)

The measurement of TFC was based on a previous study [27]. The extracts were dissolved in 95% methanol at a concentration of 25 mg/mL, and standard solutions were prepared. 50 μL of each extract or standard was added sequentially with 350 μL of 99.8% methanol, 20 μL of 10% AlCl_3_, and 20 μL of 1.0 M potassium acetate, followed by 500 μL of water. After thoroughly mixing and incubating in the dark for 30 min, 200 μL of the mixture was transferred to a 96-well microplate. The absorbance was measured at 415 nm using a microplate reader (BioTek) and the TFC of the extracts was calculated using the standard curve equation of quercetin to express as quercetin equivalents (QE) per gram of dried extract.

### 2.5. High Performance Liquid Chromatography (HPLC) of Major Compounds

The composition of the major compound was analyzed using HPLC equipped with a pump (PU-2080 Plus, JASCO, Tokyo, Japan), UV/Vis detector (UV-2075 Plus, JASCO), and autosampler (AS-2055 Plus, JASCO). The flow rate was 0.7 mL/min, and the injection volume was 20 μL into an Agilent C18 column (4.6 × 250 mm, 5 μm) (Santa Clara, CA, USA). The preparation of standard and sample solutions involved using 100% methanol and filtration through a 0.22 μm membrane filter. The analytical conditions were as follows: (1) Flavonoid compounds were analyzed using a gradient elution method with mobile phase solutions A (0.5% acetic acid in water) and B (100% acetonitrile). The analysis took a total of 70 min and the measurement wavelength was 260 nm [28]. (2) Phenolic compounds were analyzed using a gradient elute method using mobile phase solutions A (1.0% acetic acid in water) and B (100% methanol). The total analysis lasted 90 min and the measurement wavelength was 280 nm [29].

### 2.6. Culture of JB6 P+ Cells

JB6 P+ mouse epidermal cells were obtained from the American Type Culture Collection (ATCC, Manassas, VA, USA; catalog no. CRL-2010) and used as a model for tumor promoter-induced transformation. MEM was used to culture JB6 P+ cells together with 5% fetal bovine serum (FBS) and 1% penicillin-streptomycin. At 37 °C, the cells were maintained in a humidified 5% CO_2_ atmosphere. To store for long-term preservation, the cells were suspended in MEM containing 10% dimethyl sulfoxide (DMSO), transferred to cryovials, and stored in liquid nitrogen tanks.

### 2.7. Cell Viability Assay

At a density of 5 × 10^3^ cells/100 μL per well, JB6 P+ cells were cultured in MEM that contained 5% FBS in a 96-well plate and for a 24 h period. After removing the culture medium, cells were treated with CDL-WE or CDL-95EE dissolved in MEM containing 1% FBS. CDL-95EE was first dissolved in DMSO to prepare a stock solution, and then diluted to a final DMSO concentration of 0.01% in the medium, which does not affect cell viability. The MTS assay kit was used to measure cell viability after 72 h of incubation. The MTS reagent and culture medium were combined in a 1:5 ratio to create the detection solution. The detection solution was added to each well, and the plate was incubated at 37 °C in the dark for approximately 2 h. The absorbance was then measured at 490 nm using a microplate reader (BioTek) to calculate cell viability.

### 2.8. TPA-Induced JB6 P+ Cell Transformation Assay

Following the method described in a previous study [30], TPA-induced transformation of JB6 P+ cells was assessed in a soft agar assay. A 0.5% agar in basal medium Eagle (BME) was prepared on a 6-well plate and left to solidify at room temperature for 1 h. The top layer consisted of 0.3% agar and 8 × 10^3^ JB6 P+ cells per well after that. Cells were treated with 20 ng/mL TPA and various concentrations of CDL-WE or CDL-956EE, and incubated at 37 °C for 14 days. ImageJ software (version 1.54d, NIH, Bethesda, MD, USA) was utilized to count the number of cell colonies.

### 2.9. ROS Level Assay

To assess intracellular oxidative stress, DCFH-DA staining was used and followed by flow cytometry to measure the mean fluorescence intensity (MFI) [31]. JB6 P+ cells were seeded in 6 cm dishes at a density of 1 × 10^5^ cells/3.5 mL/dish using MEM supplemented with 5% FBS. After 24 h of incubation, the medium was replaced with 3.5 mL of MEM containing 1% FBS and various concentrations of CDL-95EE. Cells were incubated for 48 h, the medium was removed, and cells were treated with 20 ng/mL TPA and different concentrations of CDL-95EE for another 48 h. The cells were treated by washing with 1 mL PBS, then incubating with 300 μL trypsin for 5 min. Cells were collected after adding 500 μL of PBS, then centrifuged at 400× *g* for 5 min. After removing the supernatant, 200 μL of PBS that included DCFH-DA was added, and the cells were kept in the dark at 37 °C for 30 min. The cells were centrifuged at 400× *g* for 5 min, washed twice with 500 μL PBS, and centrifuged again for 5 min. The cell pellet was resuspended in 500 μL PBS, and fluorescence intensity was measured by flow cytometry (Guava EasyCyte 6-2L, Luminex Corporation, Austin, TX, USA) with excitation at 485 nm and emission at 535 nm.

### 2.10. mRNA Expression Determination

The density of JB6 P+ cells was 5 × 10^5^ cells per dish in 10 cm dishes using MEM and 5% FBS. After 24 h of incubation, the culture medium was removed and replaced with MEM containing 1% FBS and varying concentrations of CDL-95EE. Cells were incubated for another 48 h, then scraped off with 1 mL PBS after removing the culture medium. Cells were obtained by centrifuging at 400× *g* at 4 °C for 5 min, then washed twice with PBS. The collected cells were used to prepare DNA, RNA, and protein samples for further experiments. According to the manual, total RNA was extracted using the total RNA isolation kit (GeneDireX, Las Vegas, NV, USA). The RNA concentration was then determined using a spectrophotometer (Nabi, MicroDigital, Seoul, Republic of Korea). CDNA synthesis was performed using the iScript™ cDNA Synthesis Kit (Bio-Rad, Hercules, CA, USA). Nuclease-free water, iScript reaction mix, iScript reverse transcriptase, and 500 ng of RNA template were combined to create the reaction mixture that produces first-strand cDNA through reverse transcription. The iQ SYBR Green Supermix kit (Bio-Rad) was used to perform quantitative PCR (qPCR) using a CFX Connect™ Real-Time PCR Detection System (Bio-Rad). Optimized thermal cycling conditions were used to perform the qPCR reaction containing SYBR green reagent, primers, and 100 ng of first-strand cDNA. The analysis of relative mRNA expression levels targeted genes such as Nrf2, HO-1, UGT1A1, and NQO1.

### 2.11. Protein Determination

Cell pellets were lysed by resuspension in a lysis buffer containing phenylmethylsulfonyl fluoride and protease inhibitor. Cell lysates were homogenized using an ultrasonic cell disruptor (Ultrasonic 250, Hoyu Inc., Taipei, Taiwan) and then centrifuged at 17,000× *g* at 4 °C for 30 min. The supernatant was collected for protein quantification using the bicinchoninic acid (BCA) assay, with bovine serum albumin (BSA) as standard. A loading dye containing SDS was used to mix 20 μg of protein samples, then heat them at 95 °C for 5 min. After the samples were placed in the wells of an SDS-polyacrylamide gel and electrophoresed, the separated proteins were transferred onto a PVDF membrane. The membrane was sequentially incubated in a Tris-buffered solution containing 0.1% Tween-20 and primary and secondary antibodies. Finally, the protein bands were captured by detecting chemiluminescence on the PVDF membrane using the ChemiDoc imaging system (Bio-Rad). The images were used to evaluate protein expression levels using specific antibodies. Primary antibodies used were anti-Nrf2 (ABclonal, #A0674) (Woburn, MA, USA), anti-HO-1 (Proteintech, #10701-1-AP) (Rosemont, IL, USA), anti-UGT1A (GeneTex, #GTX114131) (Irvine, CA, USA), anti-DNMT1 (ABclonal, #A19679), anti-DNMT3a (ABclonal, #A2065), anti-HDAC1 (ABclonal, #A0238), and anti-HDAC4 (GeneTex, #GTX110231). β-actin (GeneTex, #GTX109639) was used as loading controls. The membranes were incubated with primary antibodies for 4 °C overnight, and then HRP-conjugated secondary antibodies (anti-rabbit IgG, GeneTex, #GTX213110-01) were used at room temperature for 1 h. Densitometric analysis using ImageJ software (version 1.54d, NIH) was used to quantify Western blot bands and calculate the relative protein expression by comparing target protein intensity to β-actin ratio.

### 2.12. Quantitative Methylation-Specific PCR (qMSP)

According to a previous study [29], the Genomic DNA Isolation Kit (GeneDireX) was used to extract genomic DNA from cells. Bisulfite conversion was assessed using the EZ DNA Methylation-Lightning kit (Zymo Research, Los Angeles, CA, USA) to determine DNA methylation. qMSP was performed to analyze the methylation status of the Nrf2 promoter using the Bio-Rad iQ SYBR Green Supermix kit. DNA quantification was standardized using whole bisulfite-modified DNA as a reference, and the relative amounts of unmethylated DNA were determined using a real-time PCR detection system (Bio-Rad). The relative amount of unmethylated DNA was first normalized to whole bisulfite-modified reference DNA. The percentage of unmethylated Nrf2 promoter DNA was then calculated as unmethylated DNA/(unmethylated DNA + methylated DNA) × 100% for each sample.

### 2.13. Statistical Analysis

Data were expressed using mean ± standard deviation (SD). One-way analysis of variance (ANOVA) followed by Duncan’s new multiple range test was performed in SAS (version 9.4, SAS Institute Inc., Cary, NC, USA) to determine significant differences among mean values; statistical significance was considered at *p* < 0.05.

## 3. Results

### 3.1. CDL Are Rich in Bioactive Flavonoids and Phenolics

The objective of this study was to identify the key bioactive compounds in CDL using solvents of varying polarities to enhance extraction efficiency and to link chemical composition to bioactivity. First, this study aimed to investigate the active compounds in CDL. Extracts were produced by grinding leaves into a powder, freeze-drying them, and extracting with five solvents: hot water extract (WE), 95% ethanol extract (95EE), 70% ethanol extract (70EE), methanol extract (ME), and ethyl acetate extract (EAE). As shown in Table 1, CDL-WE exhibited the highest extraction yield (approximately 32.93%), followed by CDL-ME (approximately 23.95%), CDL-95EE (approximately 21.70%), and CDL-70EE (approximately 20.09%), while CDL-EAE showed the lowest extraction yield (approximately 7.44%). TPC varied significantly in some extracts (*p* < 0.05). CDL-WE had the highest TPC (60.81 ± 3.42 mg GAE/g), followed by CDL-70EE (58.55 ± 6.76 mg GAE/g) and CDL-ME (57.52 ± 2.38 mg GAE/g), while CDL-EAE had the lowest TPC (10.38 ± 2.22 mg GAE/g). However, TFC did not differ significantly among the extracts, with values ranging from 9.51 ± 0.78 mg QE/g (CDL-WE) to 12.33 ± 2.30 mg QE/g (CDL-95EE). Based on the results above, the variations in extraction yield, TPC, and TFC observed across various solvent extractions could be primarily influenced by solvent polarity and the chemical composition of the extracted compounds. Water- and ethanol-based extractions were the most efficient for extracting phenolic compounds, as previously investigated for their solubility [32]. CDL may have fewer hydrophobic polyphenols, as suggested by the low phenolic and flavonoid content in CDL-EAE.

**Figure 1:**
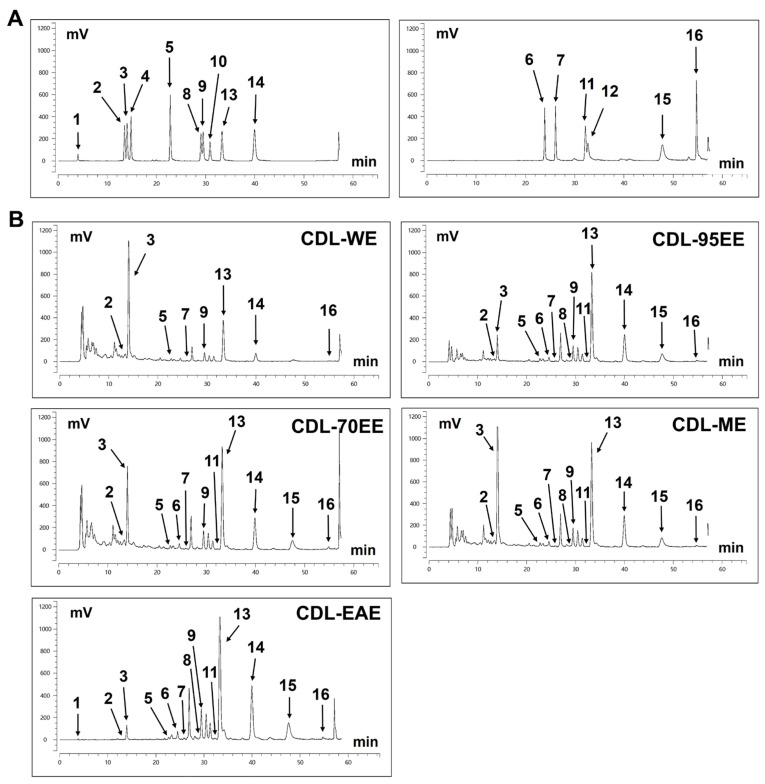
HPLC chromatograms of flavonoid compounds in different solvent extracts of CDL. (**A**) Chromatogram of standard compounds. (**B**) Chromatograms of CDL extracts obtained using different solvents: CDL-WE, CDL-95EE, CDL-70EE, CDL-ME, and CDL-EAE. Notable peaks include (1) synephrine at 4.03 min, (2) naringin at 13.52 min, (3) hesperidin at 14.04 min, (4) neohesperidin at 14.83 min, (5) naringenin at 22.81 min, (6) 7-hydroxy-3′,4′,5,6,8-pentamethoxyflavone at 23.90 min, (7) 4′-hydroxy-5,6,7,8-tetramethoxyflavone at 26.09 min, (8) 3′,4′,5,7-tetramethoxyflavone at 29.06 min, (9) sinensetin at 29.50 min, (10) 3′,4′-dimethoxyflavone at 30.91 min, (11) 3′,4′,5′,5,6,7-hexamethoxyflavone at 32.15 min, (12) 4′,5-dihydroxy-6,7,8-trimethoxyflavone at 32.66 min, (13) nobiletin at 33.34 min, (14) tangeretin at 39.95 min, (15) 5-hydroxy-3′,4′,6,7,8-pentamethoxyflavone at 47.78 min, and (16) 5-hydroxy-4′,6,7,8-tetramethoxyflavone at 54.75 min.

To determine the composition and content of potential bioactive flavonoid compounds in different CDL extracts, HPLC analysis was performed using standard compounds, as shown in Figure 1. The quantitative analysis of the main flavonoid compounds is detailed in Table 2. The main flavonoids and PMFs detected include synephrine at 4.03 min, naringin at 13.52 min, hesperidin at 14.04 min, naringenin at 22.81 min, sinensetin at 29.50 min, nobiletin at 33.34 min, tangeretin at 39.95 min, and 5-hydroxy-3′,4′,6,7,8-pentamethoxyflavone at 47.78 min. Several other methoxyflavones and various hydroxylated derivatives were also detected in different extracts. According to Table 2, CDL-WE contained the highest amount of synephrine (65.60 ± 4.59 mg/g), followed by hesperidin (37.17 ± 6.10 mg/g) and naringin (20.12 ± 0.25 mg/g). In addition to synephrine (38.75 ± 7.75 mg/g), CDL-95EE had a higher amount of nobiletin (26.07 ± 4.88 mg/g) and tangeretin (7.06 ± 1.46 mg/g). CDL-70EE and CDL-ME also had high levels of synephrine, naringin, and nobiletin, and CDL-EAE mainly consisting of nobiletin (53.37 ± 12.99 mg/g) and tangeretin (14.96 ± 3.89 mg/g) in the main component.

**Figure 2:**
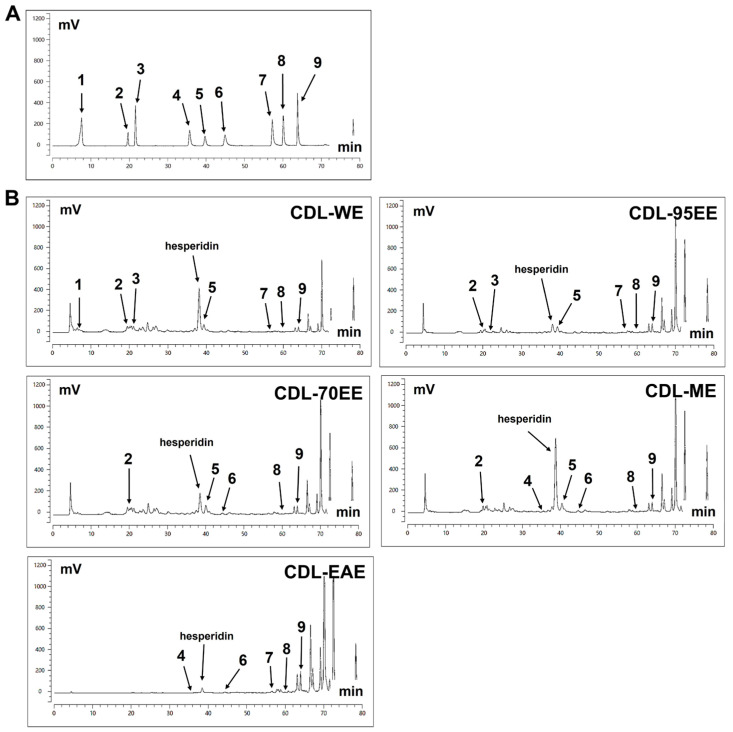
HPLC chromatograms of phenolic compounds in different solvent extracts of CDL. (**A**) Chromatogram of standard compounds. (**B**) Chromatograms of CDL extracts obtained using different solvents: CDL-WE, CDL-95EE, CDL-70EE, CDL-ME, and CDL-EAE. Notable peaks include (1) gallic acid at 7.60 min, (2) catechin at 19.68 min, (3) caffeic acid at 21.61 min, (4) myricitrin at 35.72 min, (5) rutin at 39.72 min, (6) myricetin at 44.91 min, (7) quercetin at 57.21 min, (8) luteolin at 60.09 min, and (9) apigenin at 63.817 min.

The phenolic levels of various CDL extracts were further evaluated, and Figure 2 shows the chromatograms of the standards and extracts. The results confirm the presence of key bioactive phenolics detected: gallic acid at 7.60 min, catechin at 19.68 min, caffeic acid at 21.61 min, myricitrin at 35.72 min, rutin at 39.72 min, myricetin at 44.91 min, quercetin at 57.21 min, luteolin at 60.09 min, and apigenin at 63.82 min. In Table 3, it was revealed that rutin was the main phenolic compound present in most extracts, with the highest concentration found in CDL-70EE (6.05 ± 0.28 mg/g), followed by CDL-ME (5.57 ± 0.56 mg/g), CDL-WE (4.62 ± 0.33 mg/g), and CDL-95EE (4.51 ± 0.73 mg/g). The high concentration of apigenin (2.71 ± 0.57 mg/g) in CDL-EAE suggests a unique bioactive profile compared to the rutin-rich extracts.

Taken together, these data indicate that solvent polarity significantly influences the extraction yield and the relative abundance of key bioactive constituents in CDL. CDL-WE provided the highest extraction yield and total phenolic content, whereas CDL-95EE was particularly enriched in total flavonoids and PMFs, including nobiletin and tangeretin, that have been linked to Nrf2 activation and chemopreventive effects. Additionally, CDL-WE and CDL-95EE exhibited low cytotoxicity against JB6 P cells (IC_50_ > 80 μg/mL). Based on their extraction efficiency, phytochemical composition, and safety, CDL-WE and CDL-95EE were selected as the most suitable candidates for subsequent mechanistic studies.

### 3.2. CDL-95EE Mitigates TPA-Induced Oxidative Stress and Inhibits JB6 P+ Cell Transformation

**Figure 3:**
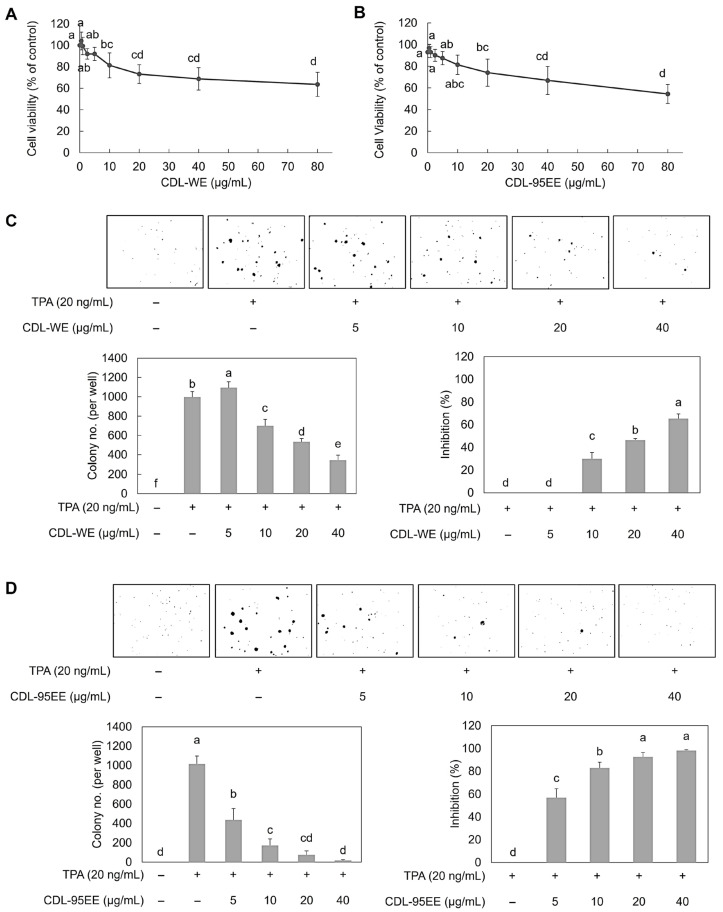
Effects of CDL-WE and CDL-95EE on JB6 P+ cell viability and TPA-induced cell transformation. (**A**,**B**) JB6 P+ cells were treated with different concentrations of CDL-WE and CDL-95EE, respectively, and cell viability was evaluated using the MTS assay. (**C**,**D**) JB6 P+ cells were treated with TPA to induce transformation and treated with different concentrations of CDL-WE and CDL-95EE. Data are expressed as mean ± SD (*n* = 3). Data with different letters (a, b, c, d, e) indicate statistically significant differences between groups (*p* < 0.05).

The potential of CDL extracts to protect against cancer was assessed in vitro using JB6 P+ cells by measuring cytotoxicity, antioxidant activity, and inhibition of tumor promoter-induced transformation. The aim of these experiments was to determine whether CDL-WE and CDL-95EE, which are rich in bioactive flavonoids, can reduce oxidative stress and prevent early tumorigenic events. The cytotoxic and cancer chemopreventive potentials of CDL-WE and CDL-95EE in normal mouse skin JB6 P+ cells were evaluated, and the results are shown in Figure 3. Figure 3A,B indicate that both CDL-WE and CDL-95EE exhibit a significant dose-dependent decrease in cell viability, respectively. Low cytotoxicity was indicated by IC_50_ values greater than 80 µg/mL, indicating that these extracts are suitable for further experimentation without causing cell damage. To further assess the cancer chemopreventive potential of CDL extracts, we examined the growth of TPA-induced anchorage-independent cells (Figure 3C,D). The control group showed minimal colony formation, whereas treatment with TPA (20 ng/mL) led to a significant increase in colony formation, confirming successful transformation. CDL-WE (10–40 µg/mL) and CDL-95EE (5–40 µg/mL) had a significant decrease in colony formation (*p* < 0.05) due to their dose-dependent inhibitory effect on TPA-induced transformation. CDL-95EE, unlike CDL-WE, showed stronger inhibition, reducing TPA-induced JB6 P+ cell viability by 50%, making it a promising candidate for studying its protective mechanisms.

**Figure 4:**
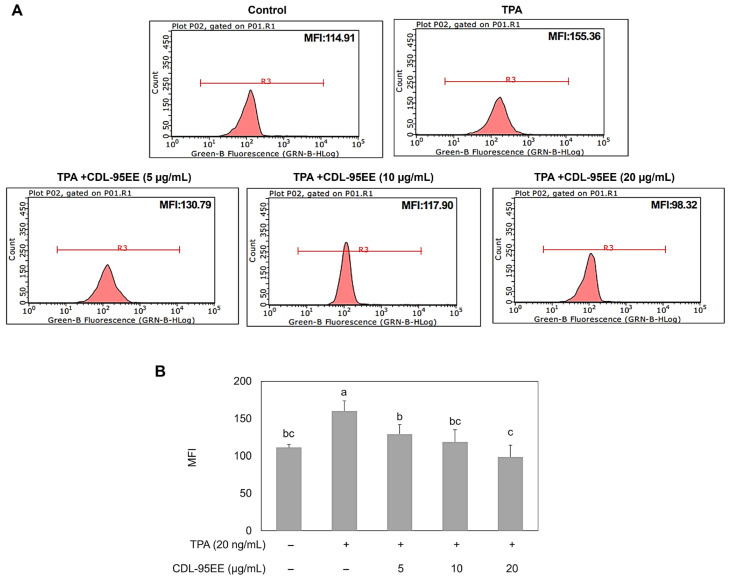
Effect of CDL-95EE on TPA-induced ROS generation in JB6 P+ cells. (**A**) Flow cytometry histograms showing ROS levels in JB6 P+ cells under different treatments. The mean fluorescence intensity (MFI) was measured to assess intracellular ROS levels. (**B**) Quantitative analysis of ROS levels in JB6 P+ cells treated with TPA and different concentrations of CDL-95EE. Data are expressed as mean ± SD (*n* = 3). Data with different letters (a, b, c) indicate statistically significant differences between groups (*p* < 0.05).

The correlation between CDL-95EE chemopreventive properties and antioxidant potential was confirmed by measuring intracellular ROS levels via flow cytometry with DCFH-DA staining following TPA treatment (Figure 4). TPA caused a significant increase in mean fluorescence intensity (MFI) to 155.36, indicating elevated oxidative stress in JB6 P+ cells. The dose range of CDL-95EE (5–20 µg/mL) resulted in a decrease in MFI, with the lowest value observed at 20 µg/mL, indicating its potent antioxidant properties.

### 3.3. CDL-95EE Activates the Nrf2 Pathway Through Transcriptional and Epigenetic Regulation

**Figure 5:**
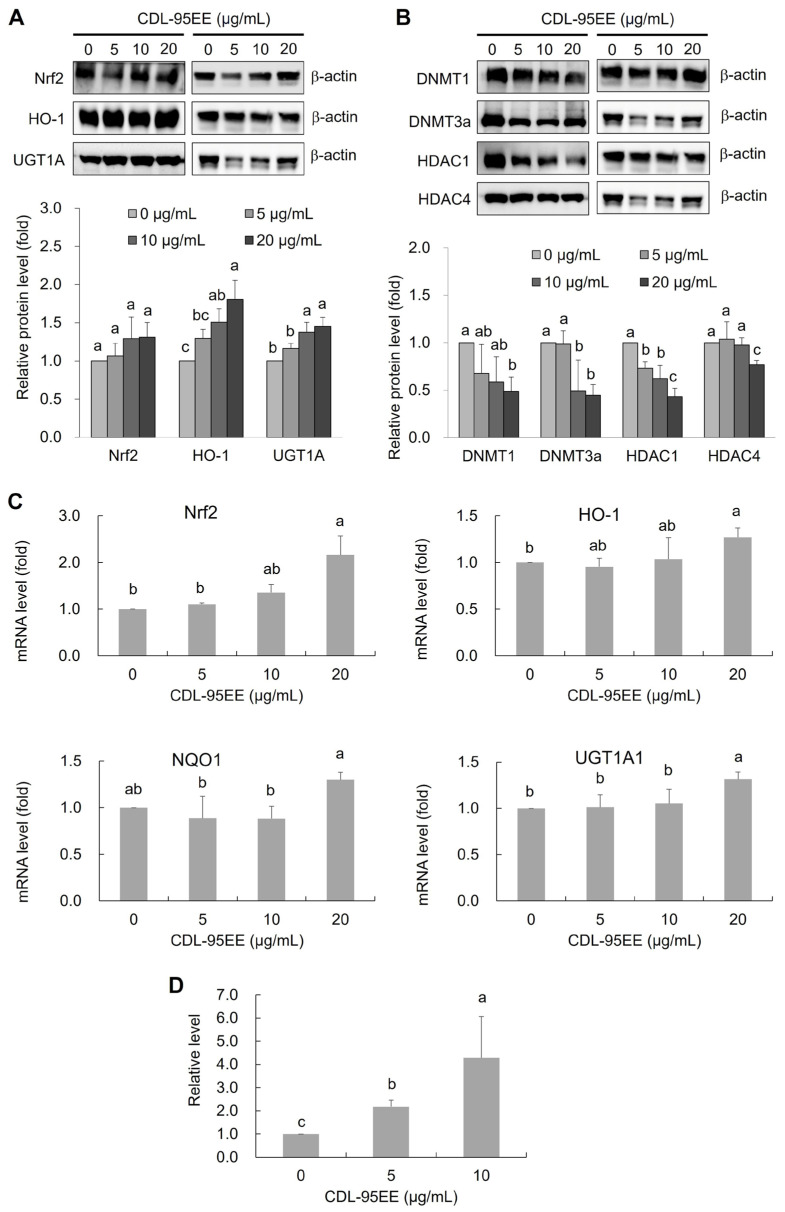
Effect of CDL-95EE on Nrf2 pathway activation and epigenetic modifications in JB6 P+ cells. (**A**,**B**) Western blot analysis of Nrf2 pathway-related antioxidant and detoxification enzymes (HO-1, UGT1A1, NQO1) and epigenetic regulators (DNMTs, HDACs) following CDL-95EE treatment, respectively. Representative Western blots for each protein and the corresponding β-actin loading control from the same experiment are shown. (**C**) Quantitative analysis of mRNA expression levels of HO-1, UGT1A1, and NQO1 normalized to β-actin. (**D**) Quantitative methylation-specific PCR (qMSP) analysis of Nrf2 promoter unmethylation levels after treatment with CDL-95EE. Data are expressed as mean ± SD (*n* = 3). Data with different letters (a, b, c) indicate statistically significant differences between groups (*p* < 0.05).

Our study demonstrated that CDL-95EE has antioxidant and anti-transformative properties, so we examined the regulatory pathways of Nrf2 signaling, a pathway that is frequently suppressed in cancer. The importance of Nrf2 in preventing oxidative stress has been demonstrated [4], and our study explored whether CDL-95EE increases Nrf2 expression and the downstream antioxidant enzymes through transcriptional activation and epigenetic regulation (Figure 5). The expression of Nrf2 and its downstream antioxidant enzymes, including HO-1 and UGT1A1, was examined by Western blotting after treatment with CDL-95EE at various concentrations. As shown in Figure 5A, CDL-95EE significantly increased Nrf2 protein levels in a dose-dependent manner, indicating that CDL-95EE may function as an activator of Nrf2. Treatment with CDL-95EE activated the Nrf2 pathway, as indicated by increased expression of HO-1 and UGT1A1, two key enzymes involved in detoxification and cellular protection against oxidative stress.

To further explore how CDL-95EE activates Nrf2, we investigated the expression levels of DNA methyltransferases (DNMTs) and histone deacetylases (HDACs), which are key regulators of gene silencing (Figure 5B). Treatment with CDL-95EE resulted in a dose-dependent decrease in the levels of the DNMT1 and DNMT3a proteins. DNMT1 is recognized for maintaining DNA methylation patterns, while DNMT3a is responsible for de novo methylation [8]. Their suppression indicates that CDL-95EE may prevent hypermethylation of the Nrf2 promoter, a common mechanism of Nrf2 silencing in cancer cells [33]. However, HDACs can deacetylate histone proteins, leading to chromatin condensation and transcriptional repression. HDAC1 and HDAC4 decreased significantly in a dose-dependent manner, indicating that CDL-95EE increases chromatin accessibility, which could lead to activation of the Nrf2 pathway.

To verify whether the increase in protein levels was transcriptionally controlled, qPCR was performed to analyze Nrf2 expression and mRNA levels of its downstream enzyme after treatment with CDL-95EE (Figure 5C). The results demonstrate a significant increase in Nrf2, HO-1, and UGT1A1 mRNA expressions in cells treated with 20 µg/mL of CDL-95EE, which is consistent with the results of Western blot. To verify that CDL-95EE-mediated Nrf2 activation is a result of epigenetic regulation, we performed qMSP to examine the methylation status of the Nrf2 promoter (Figure 5D). CDL-95EE increased the proportion of unmethylated Nrf2 promoter DNA in a dose-dependent manner. The relative level of unmethylated promoter increased from approximately 1-fold in the control group to 2.18- and 4.28-fold at 5 and 10 µg/mL CDL-95EE, respectively (*p* < 0.05), indicating progressive demethylation of the Nrf2 promoter region. This finding suggests that the potential chemopreventive effects of CDL-95EE are highlighted because it can demethylate Nrf2, which is often silenced by hypermethylation in cancer and oxidative stress-related diseases. Previous studies have also shown that PMFs, such as nobiletin and tangeretin, increase Nrf2 activation and regulate detoxification enzymes [34,35]. Some flavonoids have been shown to modulate DNA methylation and gene expression, thereby conferring long-term protection against oxidative stress [12]. Since Nrf2 silencing due to promoter hypermethylation is frequently observed in tumorigenesis, restoration of Nrf2 expression by CDL-95EE, rich in PMFs and flavonoids, may contribute to its cancer prevention properties.

## 4. Discussion

The composition of flavonoids and phenolic compounds in CDL extracts is strongly influenced by solvent polarity, which directly affects extraction efficiency and the resulting biological potential. Ethyl acetate extraction (CDL-EAE) was more effective in extracting lipophilic compounds, particularly nobiletin, tangeretin, and apigenin, compared to water and ethanol extractions (CDL-WE, CDL-70EE, and CDL-ME) that produced higher levels of hydrophilic flavonoids such as synephrine, hesperidin, naringin, rutin, and catechin. Nobiletin and tangeretin, both found in CDL, have been extensively researched for their anti-inflammatory, antioxidant, and anticancer activities [36,37]. These compounds have been shown to modulate oxidative stress and inflammation by activating the Nrf2 pathway, which is crucial for regulating cellular defense mechanisms against oxidative damage [5]. CDL-95EE and CDL-EAE have high levels of nobiletin and tangeretin, suggesting strong chemopreventive potential and making them promising candidates for future research in cancer prevention. Synephrine, the main alkaloid in CDL-WE and CDL-70EE, has been shown to enhance thermogenesis and metabolic activity [35], suggesting that these extracts could be used in nutraceuticals and functional foods to improve metabolic health. In addition, the most abundant phenolic compound in CDL-70EE and CDL-ME was rutin, which has been extensively studied for its antioxidant, anti-inflammatory, and anticancer properties [38]. Apigenin, enriched in CDL-EAE, is a flavone with potential chemopreventive activity, owing to its ability to modulate apoptosis and inhibit tumorigenesis via epigenetic mechanisms [39]. These findings highlight how solvent selection plays a crucial role in maximizing bioactive compound recovery and customizing CDL extracts for specific health applications. To further advance this field, it will be important to systematically evaluate the biological functions of CDL extracts, particularly their ability to regulate oxidative stress and suppress early carcinogenic events.

We found that CDL-95EE contained high levels of PMFs in this study, particularly nobiletin and tangeretin, which have been widely recognized for their anti-inflammatory, antioxidant, and anticancer properties [36,37]. Some PMFs have also been shown to activate the Nrf2 pathway, a central regulator of antioxidant defense [34,35]. These results indicate that CDL-95EE is a highly effective natural chemopreventive agent that reduces oxidative stress and blocks TPA-induced transformation in JB6 P+ cells. PMFs, including nobiletin and tangeretin, have been reported to enhance the Nrf2 signaling pathway by various mechanisms, including facilitating Nrf2 nuclear translocation, preventing Keap1-Nrf2 binding, and boosting ARE-driven gene transcription [40,41]. Moreover, various citrus flavonoids, such as hesperetin and naringenin, can modulate epigenetic regulators, including DNMTs and HDACs, thereby facilitating Nrf2 reactivation in cancer cells [42,43]. These findings support the notion that the chemopreventive effects of CDL-95EE are, at least in part, attributable to its rich PMF/flavonoid profile and the activation of the Nrf2 antioxidant pathway through combined transcriptional and epigenetic mechanisms. To confirm the causal role of Nrf2 in mediating the observed effects of CDL-95EE, further studies could use Nrf2-specific inhibitors or siRNA-mediated knockdown.

There are several limitations to this study. First, the experiments were done in a single in vitro model using murine JB6 P skin epidermal cells, but there is no evidence of the chemopreventive effects of CDL-WE or CDL-95EE in vivo. The bioavailability, metabolism, tissue distribution, and safety of CDL extracts, as well as their chemopreventive efficacy, need to be evaluated in appropriate animal models and carefully planned clinical experiments in the future. Second, although our data demonstrate robust activation of Nrf2 and its downstream targets, along with decreased DNMT and HDAC expression and reduced Nrf2 promoter methylation, we did not perform functional inhibition of Nrf2 (e.g., siRNA knockdown or pharmacological inhibitors such as ML385) and thus cannot fully establish causality. Finally, our epigenetic analysis was limited to the Nrf2 promoter and did not include global DNA methylation or broader genome-wide epigenetic changes. Future investigations should therefore include Nrf2 loss-of-function approaches and genome-wide epigenetic profiling, as well as isolation and characterization of individual CDL components, to identify the key molecules driving Nrf2 reactivation and long-term chemopreventive effects.

## 5. Conclusions

The results of this study show that CDL extracts, particularly CDL-95EE, exhibit strong antioxidant and chemopreventive properties. CDL-95EE, which contains nobiletin and tangeretin, effectively reduces oxidative stress, inhibits the transformation of JB6 P+ cells, and activates the Nrf2 pathway. Our findings revealed that CDL-95EE enhances Nrf2 expression by regulating epigenetic mechanisms, including downregulation of DNMT1, DNMT3a, HDAC1, and HDAC4, and demethylation of the Nrf2 promoter, leading to increased transcription of the antioxidant enzymes HO-1 and UGT1A1. These findings indicate that CDL-95EE could be a potential natural agent for cancer prevention. However, since the current results are based solely on in vitro models, more in vivo or clinical investigations are required before evaluating their use in functional foods or nutraceuticals.

## Figures and Tables

**Table 1:** Extraction yield, total phenolic content, and total flavonoid content of different CDL extracts ^1^.

	CDL-WE	CDL-95EE	CDL-70EE	CDL-ME	CDL-EAE
Extraction yield (g/100 g dried material) ^2^	32.93 ± 1.50 ^a^	21.70 ± 1.05 ^bc^	20.09 ± 2.09 ^c^	23.95 ± 1.60 ^b^	7.44 ± 0.77 ^d^
Total phenolic content (mg GAE/g dried extract) ^3^	60.81 ± 3.42 ^a^	25.99 ± 3.71 ^b^	58.55 ± 6.76 ^a^	57.52 ± 2.38 ^a^	10.38 ± 2.22 ^c^
Total flavonoid content (mg QE/g dried extract) ^4^	9.51 ± 0.78 ^a^	12.33 ± 2.30 ^a^	10.2 ± 0.40 ^a^	12.06 ± 0.99 ^a^	10.5 ± 2.65 ^a^

^1^ Data are expressed as mean ± SD (*n* = 3). A statistically significant difference (*p* < 0.05) can be observed in the values of the same measurement item with different superscript letters (a, b, c, d). ^2^ Extraction yield (%) = [Weight of freeze-dried extract (g)/Weight of dried powder (g)] × 100%; ^3^ GAE: gallic acid equivalent; ^4^ QE: quercetin equivalent.

**Table 2:** Major flavonoid compounds identified in different CDL extracts ^1^.

Peak No.	Compound	Content (mg/g Dried Extract)				
		CDL-WE	CDL-95EE	CDL-70EE	CDL-ME	CDL-EAE
1	Synephrine	65.60 ± 4.59 ^a^	38.75 ± 7.75 ^b^	59.49 ± 1.94 ^a^	63.19 ± 8.64 ^a^	1.55 ± 0.42 ^c^
2	Naringin	20.12 ± 0.25 ^a^	2.55 ± 0.44 ^b^	25.31 ± 2.21 ^a^	22.57 ± 5.94 ^a^	0.78 ± 0.22 ^b^
3	Hesperidin	37.17 ± 6.10 ^b^	6.05 ± 0.89 ^c^	14.19 ± 1.13 ^b^	77.10 ± 26.17 ^a^	3.38 ± 1.20 ^c^
4	Neohesperidin	N.D.	N.D.	N.D.	N.D.	N.D.
5	Naringenin	0.15 ± 0.02 ^c^	0.26 ± 0.06 ^b^	0.24 ± 0.05 ^bc^	0.39 ± 0.06 ^a^	0.21 ± 0.06 ^bc^
6	7-Hydroxy-3′,4′,5,6,8-pentamethoxyflavone	N.D.	0.01 ± 0.00 ^b^	0.01 ± 0.00 ^b^	0.01 ± 0.00 ^b^	0.03 ± 0.01 ^a^
7	4′-Hydroxy-5,6,7,8-tetramethoxyflavone	0.03 ± 0.00 ^a^	0.04 ± 0.01 ^a^	0.04 ± 0.00 ^a^	0.05 ± 0.01 ^a^	0.33 ± 0.27 ^b^
8	3′,4′,5,7-Tetramethoxyflavone	N.D.	N.D.	N.D.	0.01 ± 0.01	0.01 ± 0.11
9	Sinensetin	3.94 ± 0.15 ^c^	6.56 ± 0.92 ^bc^	7.07 ± 1.34 ^bc^	8.54 ± 0.94 ^b^	13.74 ± 3.81 ^a^
10	3′,4′-Dimethoxyflavone	N.D.	N.D.	N.D.	N.D.	N.D.
11	3′,4′,5′,5,6,7-Hexamethoxyflavone	N.D.	0.13 ± 0.03 ^b^	0.11 ± 0.03 ^b^	0.15 ± 0.02 ^b^	0.35 ± 0.12 ^a^
12	4′,5-Dihydroxy-6,7,8-trimethoxyflavone	N.D.	N.D.	N.D.	N.D.	N.D.
13	Nobiletin	12.53 ± 0.34 ^c^	26.07 ± 4.88 ^b^	23.32 ± 0.96 ^bc^	29.4 ± 4.07 ^b^	53.37 ± 12.99 ^a^
14	Tangeretin	2.31 ± 0.12 ^a^	7.06 ± 1.46 ^b^	8.07 ± 1.49 ^b^	8 ± 1.20 ^b^	14.96 ± 3.89 ^c^
15	5-Hydroxy-3′,4′,6,7,8-pentamethoxyflavone	1.78 ± 0.20 ^c^	5.27 ± 0.78 ^b^	5.4 ± 0.97 ^ab^	6.54 ± 0.67 ^b^	10.79 ± 2.52 ^a^
16	5-Hydroxy-4′,6,7,8-tetramethoxyflavone	0.23 ± 0.07	0.10 ± 0.01	0.08 ± 0.01	0.66 ± 0.94	0.23 ± 0.07

^1^ Data are expressed as mean ± SD (*n* = 3). A statistically significant difference (*p* < 0.05) can be observed in the values of the same compound with different superscript letters (a, b, c). N.D.: Not detected.

**Table 3:** Major phenolic compounds identified in different CDL extracts ^1^.

Peak No.	Compound	Content (mg/g Dried Extract)				
		CDL-WE	CDL-95EE	CDL-70EE	CDL-ME	CDL-EAE
1	Gallic acid	0.10 ± 0.04	N.D.	N.D.	N.D.	N.D.
2	Catechin	2.57 ± 0.27 ^a^	1.75 ± 0.23 ^a^	2.67 ± 1.85 ^a^	3.19 ± 0.21 ^a^	N.D.
3	Caffeic acid	0.05 ± 0.00 ^a^	0.01 ± 0.00 ^a^	N.D.	N.D.	N.D.
4	Myricitrin	N.D.	N.D.	0.63 ± 0.02 ^a^	0.60 ± 0.04 ^a^	0.07 ± 0.01 ^b^
5	Rutin	4.62 ± 0.33 ^bc^	4.51 ± 0.73 ^c^	6.05 ± 0.28 ^a^	5.57 ± 0.56 ^ab^	N.D.
6	Myricetin	N.D.	N.D.	1.06 ± 0.01 ^b^	1.48 ± 0.26 ^a^	0.75 ± 0.06 ^c^
7	Quercetin	0.14 ± 0.01 ^a^	0.12 ± 0.01 ^ab^	N.D.	N.D.	0.10 ± 0.00 ^b^
8	Luteolin	0.13 ± 0.01 ^b^	0.16 ± 0.01 ^b^	0.14 ± 0.00 ^b^	0.18 ± 0.01 ^a^	0.21 ± 0.02 ^a^
9	Apigenin	0.77 ± 0.09 ^c^	1.33 ± 0.26 ^b^	1.11 ± 0.04 ^bc^	1.42 ± 0.11 ^b^	2.71 ± 0.57 ^a^

^1^ Data are expressed as mean ± SD (*n* = 3). A statistically significant difference (*p* < 0.05) can be observed in the values of the same compound with different superscript letters (a, b, c). N.D.: Not detected.

## Data Availability

The raw data supporting the conclusions of this article will be made available by the authors on request.
